# Broad host range may be a key to long-term persistence of bacteriophages infecting intestinal *Bacteroidaceae* species

**DOI:** 10.1038/s41598-022-25636-x

**Published:** 2022-12-06

**Authors:** Stina Hedžet, Maja Rupnik, Tomaž Accetto

**Affiliations:** 1 Department for Microbiological Research, Centre for Medical Microbiology, National Laboratory for Health, Environment and Food, NLZOH, Prvomajska Ulica 1, 2000 Maribor, Slovenia; 2 Faculty of Medicine, University of Maribor, Taborska Ulica 8, 2000 Maribor, Slovenia; 3 Department of Microbiology, Biotechnical Faculty, University of Ljubljana, Groblje 3, 1230 Domžale, Slovenia

**Keywords:** Bacteriophages, Phage biology, Microbiome

## Abstract

The longitudinal studies have found that the human gut microbiota is stable over time with some major bacterial lineages or even strains persisting for years. This was recently extended to gut bacteriophages using the metagenomic data. Here, we focused on cultivation of the major *Bacteroidetes* of human gut, the *Bacteroides* and *Phocaeicola* strains, and their bacteriophages from two healthy donors. The persistence of *Bacteroides* and *Phocaeicola* species and strains was confirmed. We isolated 28 genetically different phages grouped into seven distinct clusters, two of these were new. Moreover, the bacteriophages from several groups, although being genetically quite homogeneous, had the ability to infect the strains belonging to different species isolated from several sampling time-points and different donors. We propose that the ability to infect several host species, which differ in their nutritional niches, may promote long-term persistence of dominant gut bacteriophage groups.

## Introduction

Due to the enormous interest in gut microbiota and its influence on human health, the roles of their viral parasites/predators, the bacteriophages (phages), are also being increasingly recognized. Phages manipulate bacterial diversity and composition in intestinal microbiota through predation and horizontal gene transfer^1,2^, and the gut virome can correlate with a variety of common chronic diseases^3–6^.

Virome analyses are still challenging due to the lack of universal marker genes, deficient taxonomic classification, and small numbers of gut phages in existing databases. However, a substantial number of gut viral metagenomic assemblies with large-scale catalogues of phage genomes were published recently, which should facilitate virome exploration. These studies have also shown that the gut virome is dominated by phages that infect members of the classes *Bacteroidia* and *Clostridia*^3,7–9^ In addition to metagenomic approaches, isolating and characterizing phages remains crucial to better understand host-phage interactions, host range, and phage stability and persistence in the gut.

To date, several phage groups of predominant intestinal bacterial genera and species have been isolated. The most notable has been the isolation of five representatives of an in-silico discovered highly abundant viral clade^10^, now a newly established ordo *Crassvirales*^11^. Generally, phages from this highly abundant group are most probably unable to form stable plaques on agar lawn. Thus, their isolation is more challenging and involves cultivation in liquid media coupled with metagenomic sequencing. The first isolated CrAss-like phage, Crass001, infects *Bacteroides intestinalis*^12^ and two closely related isolates were later reported from *Bacteroides thetaiotaomicron* (DAC15 and DAC17)^13^. ΦcrAss002 was isolated from the enriched supernatant of *Bacteroides xylanisolvens*^14^. Temperate phages, with diversity-generating retroelements (DGRs), were identified in the genomes of *Faecalibacterium prausnitzii*^5^ and *Bacteroides dorei*^15^. Additionally, the following phages that infect *Bacteroidaceae* species have been isolated and sequenced: two phage clusters that infect *B. thetaiotaomicron*^13^, B40-8^16^ and B124-14^17^ that infect *Bacteroides fragilis*, ϕBrb01 and ϕBrb02 isolated from rumen fluid infecting ruminal *Bacteroides* sp.^18^ and Bacuni phages with DGRs that infect *Bacteroides uniformis*, recently isolated by our group^19^.

Less is known about the temporal dynamics of phage gut communities over longer periods of time. Here, we analyzed fecal samples obtained from unlinked individuals several months to 2 years apart. Instead of relying on purely metagenomic data and bioinformatic predictions of bacteriophage cluster tropism, we opted for cultivation approach revealing host range differences at the bacterial strain level. We report the isolation of 28 phages from seven distinct known or new clusters that infect *Bacteroides* and *Phocaeicola*. Comparison of strains and phages obtained from different donors and distinct time points revealed long-term persistence of bacterial lineages, viral clusters and pronounced cross species infectivity of obtained bacteriophages. We propose that the conserved potential to infect various species within the family *Bacteroidaceae* could contribute to the observed long-term stability of these phage groups in the gut.

## Results

**Figure 1:**
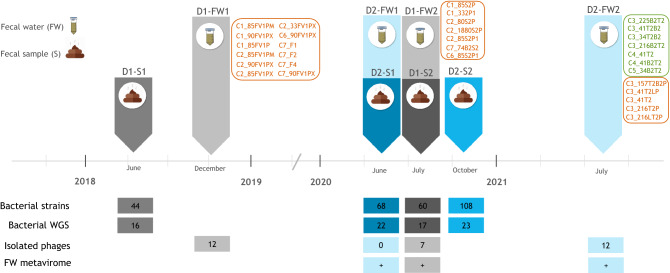
Experimental timeline. D1 and D2: donor 1 (grey) and 2 (blue); S1 and S2: first and second sampling and bacterial isolation; FW-1 and FW-2: first and second fecal water sampling, respectively. The bacteriophages are listed next to the fecal water sample where they originated. Bacteriophages in orange were isolated using plaque assays while those in green were recovered in enrichment cultures. The lower part describes the number of *Bacteroides* and *Phocaeicola* strains isolated and sequenced (WGS: whole genome sequencing), the number of isolated bacteriophages and timepoints of metavirome samples.

Fecal samples were obtained from two healthy donors at two different time points (D1 and D2, Fig. 1) and were used for bacterial and phage cultivation. The time points for bacterial and phage isolation were not always the same. The bacterial strains, bacterial potentials as phage hosts, and isolated phages were then compared within and between donors.

### *Bacteroides* and *Phocaeicola* strains predominated in the isolated gut bacteria

From both donors (D1 and D2) and both time points (S1 and S2), more than 400 bacterial isolates were subcultured, of which 280 strains were obtained in pure culture and identified by MALDI-TOF and 16S rRNA sequencing (Supplementary Table 1). A subset of 199 strains belonged to eight *Bacteroides* and two *Phocaeicola* species (Supplementary Table 1)*.* Other less commonly isolated genera were *Blautia, Dorea*, and *Bifidobacterium.*

The most abundant isolated species at all time points were *P. vulgatus, B. uniformis*, *B. ovatus*, and *B. thetaiotaomicron* (in donor D2 only)*.* Whole genome sequencing of 69 *Bacteroides* and *Phocaeicola* strains was performed (Supplementary Table 1) to confirm the identifications and explore the persistence of the strains. The obtained genomes were also examined for the presence of relevant prophages.

**Figure 2:**
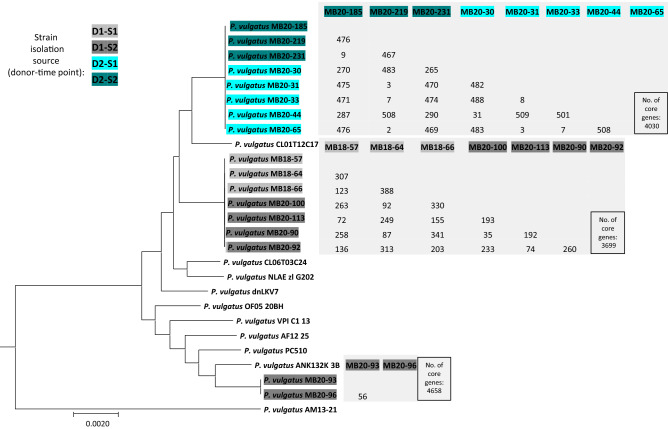
Phylogenetic tree based on core-genome alignment of *P. vulgatus* strains and genomic differences between strains clustered in one developmental line. Strains isolated from donors 1 and 2 are highlighted in grey and blue, respectively. Pairwise distances of the core genomes of bacterial strains in one developmental line is expressed as single nucleotide polymorphisms (SNPs) and shown in the corresponding matrix. Phylogenetic trees of *B. uniformis, B. ovatus* and *B. thetaiotaomicron strains* are presented in Supplementary Fig. 2.

Phylogenetic trees based on the core genomes of the four most abundant species and the number of base differences per genome pairs from each developmental line are shown in Fig. 2 and Supplementary Fig. 1. As expected, the strains isolated from donors D1 and D2 were not phylogenetically related. Strains from a given species within a single donor mostly classified into one lineage, indicating persistence in donors (ANIb ≥ 0.99). More diversity was observed in *B. uniformis* and *P. vulgatus* isolated from donor D1, as strains clustered in two different developmental lines. Within the same developmental line, strains either exhibited high similarity (2–11 SNPs) or appeared to be more phylogenetically distant (up to 687 SNPs in the core genome). The in-cluster homogeneity was highest in *B. thetaiotaomicron* (2–83 SNPs) and lowest in *B. ovatus* (2–616 SNPs) and *B. uniformis* (2–687 SNPs) (Fig. 2 and Supplementary Fig. 1).

### Isolation and genetic characterization of novel phages that infect different *Bacteroides* and *Phocaeicola* strains

Cultivated *Bacteroides* and *Phocaeicola* strains were subsequently used as potential bacterial hosts for phage isolation from the sterile fecal filtrate (fecal water) from each donor at two distinct time points (Fig. 1).

**Figure 3:**
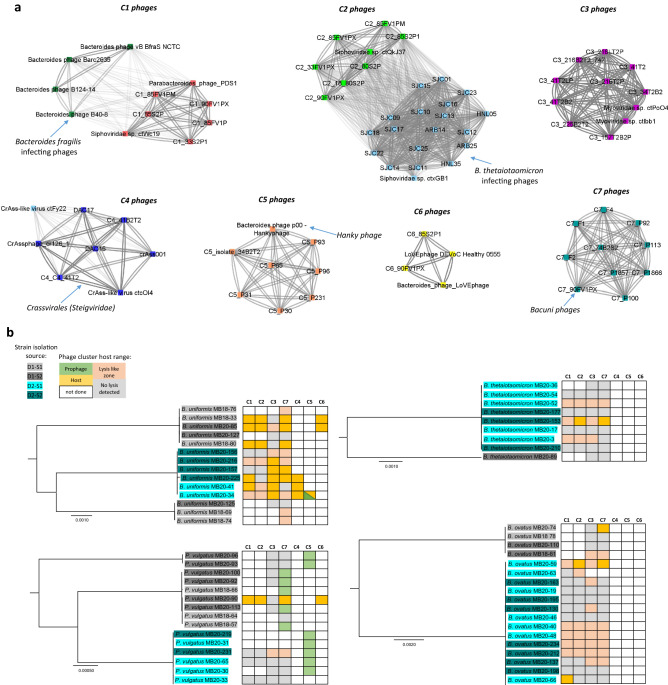
The phages isolated in this study belong to seven distinct clusters and have broad host-range. (**a**) Network taxonomic classification of isolated phage genomes, using the ViralRefSeq-prokaryotes-v94 database and known isolated *Bacteroides*-infecting phages from the literature. Only phages isolated in this study and phages related to the isolated clusters are presented. The phages are colored according to vConTACT2 cluster affiliation which corresponds to ICTV genus. Phages isolated in this study have names beginning with cluster name (C1, C2…) and prophages of C5 and C7 groups are indicated by P immediately following cluster designation. The complete output is available in Supplementary File 6. (**b**) Visualization of phage cluster host range on *B. uniformis, P. vulgatus, B. thetaiotaomicron, and B. ovatus* strains shown as phylogenetic trees based on core-genome alignment. Strains isolated from D1 and D2 are highlighted in grey and blue, respectively. Host ranges of C1, C2, C3, and C7 phages were tested with plaque and spot assays. For the explanation of lysis-like zone, please see text. For groups C4 and C5 no plaques were obtained but phages were detected using NGS sequencing in liquid cultures. Although amenable to plaque assay, no pure phage lysate could be obtained for C6, so no spot assays were done. The C5 and C7 prophages were detected using genomic analysis.

We isolated 28 genetically different phages, which vConTACT2 grouped into seven distinct clusters (C1–C7) (Fig. 3a, Supplementary Files 6, 7). Among them, the C3 phages were completely novel (Table 1, Fig. 3a) and may constitute a new bacteriophage family according to ICTV criteria^20^. C2 phages did not share any nucleotide similarity with previously described phages; however, they shared 57% protein identity (structural virion proteins) in one fifth of the genome with alpha cluster phages that infect *B. thetaiotaomicron*^13^. We propose the following cluster names: Meggiephages for C3 and Tigiphages for C2. The other clusters shared higher levels of homology with previously described or isolated phages (Table 1). C4 and C5 phages could not be propagated using plaque or spot assays and were maintained in enrichment liquid cultures (Fig. 1). Working with enrichment cultures is much more time consuming than plaque assays, as techniques like real-time PCR or sequencing must be used to confirm the presence of the phage. Thus, several tests performed for other clusters were not performed for C4 and C5.

**Table 1:** Comparison of isolated phage clusters and related phages previously described in the literature.

Phage cluster (proposed name)	Isolation source	No. genetically diverse phages	Genome size (bp)	No. of ORFs	Host species	Related phages described in literature (nucleotide identity/coverage %)	Closest BLASTn hit^31^ (identity/coverage %)	Presence in obtained metavirome
C1	D1-FW1, D1-FW2^a^	5	44,341–44,655	54–56	*B. uniformis* *P. vulgatus* *B. ovatus* *B.thetaiotamicron*	Isolated *Parabacteroides* phage PDS1 (MN929097.1), (95/90%)^41^	BK057270.1^b^; 97/88%	D1-FW2
C2 (Tigiphages)	D1-FW1, D1-FW2	7	33,659–33,821	46	*B. uniformis* *P. vulgatus* *B. ovatus* *B.thetaiotamicron*	First described and isolated in this study^c^	BK033007.1^b^; 90/92%	D1-FW2
C3 (Meggiephages)	D2-FW2	9	74,237–74,722	117	*B. uniformis*	First described and isolated in this study	BK032629.1^b^; 97/80%	D2-FW2
C4 (CrAss-like phages)	D2-FW2	2^d^	99,030–100,005	103	*B. uniformis*	Isolated CrAss-like phages ΦcrAss001^12^, ΦcrAss002^14^; DAC15 and DAC17^13^; (77/24%—ΦcrAss001)	BK035398.1^b^; 99/89%	D2-FW1, D2-FW2, D1-FW2
C5	D2-FW2	1	42,552	54	*B. uniformis* *P. vulgatus*	Isolated temperate phage of *Bacteroides dorei:* Bacteroides phage p00 Hankyphage^15^; (99/99%)	BK010646.1; 99/99%	D2-FW1, D2-FW2
C6	D1-FW1, D1-FW2	2^d^	73,875	107	*B. uniformis* *P. vulgatus*	Isolated in this study, identical to described metagenomics putative phage LoVEphage DEVoC_Healthy_0555 (MZ919987)^42^ (99.27 /94%)	MZ919987.1^b^; 99/94%	D1-FW2
C7 (*Bacuni* phages)	D1-FW1, D1-FW2	2	40,421–40,744	50–51	*B. uniformis* *P. vulgatus* *B. ovatus*	*Bacuni* phages F1, F2 and F4^19^ (99/99%)	MT635598.1; 99/99%	D1-FW2

^a^D, donor; FW, fecal water.

^b^Assembled putative viral contig from metagenomic data.

^c^App. 57% amino acid identity in 20% of ORFs with *B. thetaiotaomicron* infecting alpha cluster phages from^13^.

^d^Partial viral genomes in cluster.

In general, the genetic diversity within isolated clusters was low (Supplementary Fig. 2e). The cluster representatives were well above the 95% threshold over whole sequence for bacteriophage species^20^ and with the exception of C2, showed the nucleotide divergence of less than 0.1%. Whole genome annotations of each cluster’s phage representative are available in Supplementary Table 2 and are visualized in Supplementary Fig. 2f.

The cluster C2 was most diverse as most of the isolated phages had more than 100 SNP sites when compared to the reference phage. Additionally, deletion of the entire ORF34 gene (putative membrane protein) was detected in three representatives (Supplementary Fig. 2e).

While the C3 phages were isolated from the enrichment host cultures and further purified using double-agar-layer methods, we observed genomic divergence arising during purification passages (Supplementary Fig. 3). Six genomes, both from enrichment cultures and plaques contained an approximately 2 kbp chromosomal inversion, located in the putative tail fiber protein, which, however, did not seem to influence the host range.

From the enrichments, we obtained two complete and two partial crAss-like phage genomes (C4 phages). This is the first isolation of crAss-like phages that infect *B. uniformis*. According to the vConTACT2^21^ analysis, these phages are similar to ΦcrAss001^12^, sharing 77% nucleotide level identity in one fourth of the genome (Table 1, Fig. 3a).

Although frequently observed as prophages in the bacterial genomes in this study, only one representative of phage cluster C5 was isolated (C5_34B2T2). It was similar to the previously reported and isolated temperate phage of *B. dorei, Bacteroides* phage 00 Hankyphage^15^ (Table 1, Fig. 3a).

Further representatives of putative temperate phages were found in cluster C6, from which one whole and two additional partial genomes were isolated. These genomes were highly similar to a prevalent putative phage, identified in a Danish enteric virome catalogue, described by Van Espen and colleagues^22^ (Table 1).

We also isolated two novel *Bacuni* phages^19^, which represent cluster C7. The hot spot for SNPs in this phage cluster is in genes coding for DGRs (Table 1, Supplementary Fig. 2e and f).

### Biological characterization of isolated bacteriophages

Transmission electron microscopy revealed siphoviruses for all phage clusters except C4, which exhibited podovirus morphology. A detailed description of the isolated phages is provided in Supplementary Fig. 2a–d. Bacteriophages were obtained at different purity levels: pure phage lysate, mixed phage lysate, and pure or mixed sterile filtrate of enrichment cultures (Supplementary Fig. 2e). We were able to obtain at least one representative of the phage clusters C1, C2, C3 and C7 in pure phage lysate. Phages were stable and retained infectivity for 2 years when stored at 4 °C or − 80 °C at high concentration (10^9^ pfu/mL). C4, C5, and C6 phages were obtained as mixed sterile filtrates of enrichment cultures and mixed phage lysates respectively and for them no host range or lysogeny experiments were made.

C1 and C2 phages formed slightly turbid polymorphic plaques on supplemented Anaerobe Basal Broth (sABB) double-layer agar (Supplementary Fig. 4). The plaque size was not consistent through the purification rounds: circular plaques with diameters of 1–6 mm were formed. Plaques of C3 and C7 phages were more uniform and could be maintained with traditional double-layer agar techniques. However, C4 and C5 phages could only be propagated with enrichment culturing (Supplementary Fig. 2e).

### Certain isolated phages were detected as prophages and could be lysogenic

Phage lifestyles were deduced from genome analyses or evaluated experimentally, where dilutions of bacteria were applied on soft agar containing phages, with resultant colonies representing possible lysogens (Supplementary Table 2, Supplementary Fig. 2e,f). In C1 phages, the lysogenic gene module was not detected, and C1 phages were also unable to form stable lysogens under our experimental conditions. Lysogenic lifestyle could also not be experimentally confirmed for clusters C2 and C3. C3 phages may, however, be lysogenic since they contain lysogeny associated genes in their genomes (Supplementary Table 2, Supplementary Fig. 2e,f). The lifestyles of C4, C5, and C6 phages were not experimentally tested; however, genome and blast analysis showed that C5 and C6 phages may well be temperate, since they were both detected as prophages in multiple bacterial genomes, both in our isolated strains and genomes from NCBI nr database (Fig. 3b and Supplementary Fig. 2e). Even though temperate phages were also detected within cluster C7, since they were integrated in isolated *Phocaeicola* strain genomes, we were unable to obtain lysogens with our experimental methods in *Bacteroides* strains.

### The isolated phages are not species-specific and can infect bacterial strains isolated at various time points from both donors

Fecal water from both time points of both donors was used for phage isolation in the *Bacteroides/Phocaeicola* enrichment cultures involving the strains which again originated from both donors and time points. The strains where phages were isolated, were then designated as hosts. Additionally, purified C1 (C1-85S2P), C2 (C2-1880S2P), C3 (C3-41T2LP), and C7 (F1 and F4) phages were included in the host range experiment (spot assay) using a subset of isolated *Bacteroides* and *Phocaeicola* strains. Taken together, most of the isolated phage clusters could infect both *Bacteroides* and *Phocaeicola* species. The cumulative results derived from isolation data, spot assays, genomic analysis of isolated bacterial strains and sequencing coupled enrichment culturing are presented in the Fig. 3b. Certain phage-host combinations in spot assays resulted in bacterial lawn clearings, whereas other yielded uncharacteristic circular lysis-like zones (Supplementary Fig. 4e,f). Such opaque zones were noted before for Bacuni phages^19^ (cluster C1) and ΦcrAss002^14^. While failing to form plaques or clearings in spot assays, ΦcrAss002 was stably maintained in liquid culture and did not cause any clearing of the host bacteria^14^. It is thought that phage permissive/nonpermissive *Bacteroides* strain subpopulations arising from phase variations are the cause of this behaviour^14^. Thus, these zones indicate a possible host. In general, the spot assay did not identify many new hosts via clearing zones, but was again clearly positive with strains that were already known as positive from phage isolations. C1 and C2 phages, for example, from second time point were used in spot assays and gave clearing zones with four strains that were already known from phage isolations, produced lysis like-zones in many other strains and identified in total five additional hosts by lawn clearings. C7 (*Bacuni*) phages exhibited the broadest host range, followed by C1 and C2 phages. The novel C3 phage group tended to be more species and donor-specific. C3 phages only infected *B. uniformis* isolates and showed lysis-like zones in the other three species. Spot assay also indicated that C3 phages with or without the above mentioned 2 kb inversion could infect same hosts. For C4–C6 phages no spot assays were done. The C4 and C5 formed no plaques, while C6 were not recovered as pure phage lysates. Thus, the host designation in Fig. 3b refers to strains where they have been isolated.

**Figure 4:**
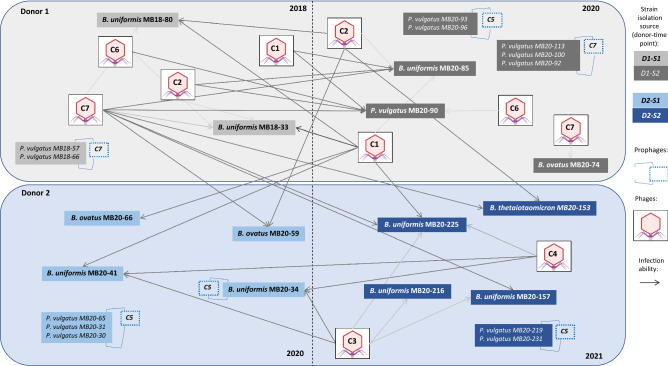
Network visualization of infection ability according to the strain source and time point shows that phages are not donor-specific. Each compartment represents an isolation time point per donor. Phages isolated at specific time points infect strains from temporally distant time points or even different donors. Prophages from the described phage clusters in this study are also shown.

Figure 4 describes presence of phage clusters in the fecal water samples and their potential to infect various hosts. C1, C2, and C7 phages were found in both time points (2 years apart) of donor D1 suggesting persistence of these phages. They could also infect *B. ovatus* and *B. thetaiotaomicron* strains from donor D2 isolated in both time-points, where these phages were not present, thus strengthening the broad host range characteristic of C1, C2 and C7 and revealing potential for spread to other human hosts. *B. uniformis* strains seemed to be most susceptible to genetically diverse phages (Figs. 3b and 4). C5 prophages were stable in *P. vulgatus* as they were retained in practically same strains isolated from donor 2 at both time points. Namely, time point 2 strain MB20-219 and strains from the first time point MB20-65 and MB20-31 contained C5 prophage and had only up to 7 SNP in more than 4000 core genes (Figs. 2 and 4) between them. For C7, this is less sure as strains harboring C7 prophages from both time points of donor 1 were not as closely related, though belonged to the same lineage of *P. vulgatus*. Interestingly, the MB20-34 strain contained a C5 prophage which was also found in filtered enrichment cultures of this strain, implying it may excise from the genome of this strain in laboratory culturing conditions.

### Metavirome sequencing provides insight into sample diversity and persistence of isolated phage clusters

Metavirome sequencing of the three fecal water samples D1-FW2, D2-FW1, and D2-FW2 confirmed the presence of the isolated viral clusters in their isolation sources while the D1-FW1 sample did not result in successful assembly. C1, C2, C6, and C7 phages were present in D1-FW2, and C3, C4, and C5 phages in D2-FW2 (Supplementary Table 3). We also evaluated virome diversity with blastn^23^ analysis (NCBI, 2022) of the largest assembled contigs (cut-off = 20 kbps) matching viral contigs identified in a recent metavirome study^3^ (Supplementary Tables 3 and 4).

Compared to the other two metaviromes, the D2-FW1 metavirome exhibited significantly lower diversity and was largely dominated by a crAss-like phage (based on k-mer assembly coverage). The CrAss-like C4 and C5 phages were present in both metaviromes of the fecal water of D2, indicating persistence of those two clusters in the gut virome (Supplementary Table 3).

## Discussion

Our longitudinal isolation and characterization of 31 phages (28 new and 3 previously published) and their corresponding *Bacteroidaceae* hosts has provided insight into persistence of bacterial hosts and phage clusters. To the best of our knowledge, this is the first longitudinal isolation of phage-host pairs from fecal samples.

We focused on *Bacteroides* and *Phocaeicola* species, Gram-negative non-spore-forming bacteria that predominate in gut microbiota and are well-adapted to the complex environment of the human gut. The stability of predominant human gut species has been reported in several metagenomics studies^24,25^. Our culturing approach combined with whole genome sequencing confirmed the persistence of bacterial gut species in humans and revealed the genetic variation of isolated *B. uniformis, P. vulgatus, B. thetaiotaomicron*, and *B. ovatus* strains. Within a single donor, they belonged to one or two developmental lines; furthermore, strain persistence was observed in all four species. Conversely, the genomes of *Bacteroides* and *Phocaeicola* strains differed significantly between donors and never belonged to the same lineage.

The phage clusters isolated in this study also persisted in individuals. For example, C1, C2, C6, and C7 phages were isolated over almost a 2-year period from the same donor, and metavirome data showed that crAss-like (C4) phages and C5 phages (similar to the *B. dorei* phage Hanky) were present in the second donor during the 1-year interval. The latter two phage groups most likely cannot form plaques and are challenging to isolate^12,14,15^. The observed persistence of our isolated phage groups are in agreement with temporal virome stability in healthy adults, as indicated by metagenomic data^26^. Our cultivation of phages also elucidated the potential mechanism for such stability: although the phages of individual groups were very similar and could not infect all strains of their original host strain lineages (Fig. 3b), they could still infect various *Bacteroides* and *Phocaeicola* species from both donors and both time points. It has been claimed that the dense gut microbiota may be an ideal place where the bacteriophage diversity can be generated since numerous strains of a single bacterial host are present and this facilitates host-range adaptation^2^. The diversity of isolated phages may thus be viewed as the end result of this process, the main finding being the broader host range than anticipated.

Not focusing on a single species may be an adaptive advantage for phages. In this way, phages can cause as little damage as possible to individual developmental lines or species and can be stably inherited even if one species diminishes in abundance or is even flushed out of the gastrointestinal tract due to dietary changes or other interventions. *Bacteroides* species have different nutritional adaptations^27^ and high genomic diversity^28^, and thus the ability of phages to infect several species may greatly contribute to their persistence within a single host and supports post-transmission establishment in new host. In this study, at least three phage clusters were capable of infecting strains from both donors (Figs. 3b and 4). The importance of host range adaptation was also implied by C5 and C7 phages that carry DGRs, which use reverse transcription to introduce nucleotide substitutions into specific target genes that may mediate host range adaptations^15,19^. Notably, most SNPs in C7 phages were found in the DGR target, the predicted phage receptor gene^19^, which is a remote homologue of *Bacteroides* fimbrial tip protein gene that may have been recruited by C7 phages long time ago^29^. On the other hand, it was shown in *B. thetaiotaomicron* that phase-variable capsular polysaccharide and lipoproteins are the main determinants of strain phage susceptibility and that they constantly change to sequentially produce phage susceptible and resistant cells^30^. It seems plausible that similar mechanisms operate in other *Bacteroides* species and yield strain subpopulations susceptible to phages that originally infected other species.

Another adaptation that could support phage transmission between individuals is a lysogenic lifestyle, and several isolated phages were also found as prophages in this study. The C7 phages, which contain both the DGR and the lysogeny genes may be an example of Piggyback-the-winner scenario phages^31^, since they may both rapidly adapt to new host bacterial strains and then lysogenize them. Of note, C7 phages were readily isolated in enrichment cultures as well as detected as prophages integrated in the host genomes.

Besides lysogeny, the above-mentioned transient phase variable phage resistance enables gut *Bacteroides* to cohabit stably with virulent phages as shown for ΦcrAss001^32^ and proposed for ΦcrAss002^14^. In our *Bacteroides* liquid enrichment cultures during phage isolation we never saw lysis and bacterial clearing indicating that Killing-the-winner^33^ scenario may be rare for *Bacteroides* phages.

In summary, we have described diverse new phages of predominant bacterial gut representatives, their long-term persistence within individual donors, and the potential to infect bacterial strains from unlinked individuals. Based on the interaction network of phage host range and isolation source (Fig. 4), we conclude that persistence and transmission potential are both enhanced by a broad host range.

## Materials and methods

### Isolation and identification of bacterial strains from human fecal samples

Fecal samples, collected from two healthy donors (D1 and D2) at two temporally distant time points (S1 and S2; four to 24 months; Fig. 1) per each donor were stored at − 80 °C. Bacuni bacteriophages from donor D1 were reported previously^19^. Fecal samples were collected together with a written informed consent and ethical approval was obtained from the National Medical Ethics Committee of the Republic of Slovenia. Samples were processed as described previously^19^. Briefly, dilutions (10^−4^ to 10^−9^) of homogenized fecal suspensions (20%) were plated on YBHI (brain–heart infusion media, supplemented with yeast extract (0.5%) and rumen fluid (20%)). After 72 h of incubation at 37 °C under anaerobic conditions, approximately 100 randomly chosen colonies per sample were subcultured on YBHI plates. Identification of pure cultures was conducted by mass spectrometry (MALDI-TOF Biotyper System, Bruker Daltonik, Bremen, Germany). The genomic DNA was isolated with QIAamp DNA Mini Kit (Qiagen, Germany) and used to PCR amplify the 16S rRNA gene using the primers 27feb to 1495revb^34^. The gene was then sequenced with the same primers and analyzed with RDP Classifier^35^. Identified pure cultures were frozen at − 80 °C in cryo-vials (Microbank, Canada). The authors confirm that all methods used were performed in accordance with the relevant guidelines and regulations.

### Phage isolation

Double-layer agar methods (plaque and spot assays) were used together with enrichment co-culturing for selected strains as described^19^ with modifications. *Bacteroides* and *Phocaeicola* strains from all four sampling points were included as potential hosts (Supplementary Table 1).

Sterile filtrate of homogenized fecal samples (fecal water; FW) from two temporally distant time points from both donors (13 to 19 months; samples D1-FW1, D1-FW2, D2-FW1, D2-FW2; Fig. 1) was prepared as described before^19^ in 50 mL of SM buffer (100 mM NaCl, 8 mM MgSO_4_, 50 mM Tris–Cl (1 M, pH 7.5) and 0.01% (w/v) gelatin). Supernatant was filtered twice through 0.2 µm pore cellulose acetate syringe membrane filters (Filtropur, Sarstedt). Fecal water was then stored at 4 °C until further use.

For enrichment co-cultures, 1 mL of host strain grown overnight in the sABB (Anaerobe Basal Broth, Thermo Fisher Scientific, supplemented with 0.12 mM MgSO_4_ and 1 mM CaCl_2_) and 1 mL of fecal water were added to 9 mL of liquid sABB and incubated for 24 h at 37 °C. Subsequently, 3 mL of culture was removed and centrifuged at 5400×*g* (4 °C). Supernatant was syringe-filtered (0.2 µm pore, Sarstedt) and added to 9 mL of fresh sABB media inoculated with the same strain as before. The procedure was repeated again after 24 h. The final sterile supernatant was refrigerated (4 °C) until further use, i.e., for the double-agar-layer method or isolation of viral DNA.

Spot assay on a double-agar-layer was used for initial phage screening. Host strains (200 µL) in the early *log* phase (OD_620_ = 0.2) were combined with 3.5 mL of soft agar (sABB) and poured onto the pre-reduced sABB agar basal plates. After solidification, tenfold dilutions of fecal water or enrichment filtrate were spotted (10 µL) onto the top agar. After overnight incubation, plates were screened for potential lysis zones. The top agar with lysis zones was harvested with an inoculation loop and stored in 100 µL of SM buffer (4 °C) overnight, followed by centrifugation (13,000×*g*, 5 min). The supernatant was then used for further steps of phage purification and characterization.

### Purification, host range, and storage of bacteriophages

Phages were initially purified from the stored spot assay supernatants by three consecutive single-plaque isolation cycles using the corresponding bacterial host strain. Top agar (3 mL, sABB) was combined with bacterial culture (200 µL) in the early *log* growth phase and diluted lysis zone supernatant and then poured onto sABB agar base plates, allowed to solidify, and incubated at 37 °C overnight. After incubation, a single plaque was picked into SM buffer (100 µL), incubated overnight (4 °C), and subsequently centrifuged (13,000×*g*, 5 min).

High-titer phage stocks were obtained from almost confluent lysed plaque assay plates by flooding the top agar overlay with 4 mL of SM buffer. Following 4 h of incubation (37 °C) with gentle shaking, the liquid phase was centrifuged at 5400×*g* (4 °C) and filter sterilized (0.2 µm pore, Sarstedt). Purified phages were stored at 4 °C and − 80 °C.

The host range of isolated phages was tested with spot assay as described above for initial phage screening using *Bacteroidaceae* strains isolated from D1 and D2 (Supplementary Table 1) and undiluted phage stocks. Transmission electron microscopy was performed by the National Institute of Biology (Ljubljana, Slovenia) as previously described^19^.

### Lysogen formation assay

Diluted stock solution (100 µL) of each isolated phage was mixed with melted sABB top agar. After solidification, tenfold dilutions of host strain in the early *log* phase were spotted on top agar. After overnight incubation (37 °C), one bacterial colony from each dilution was transferred to fresh sABB medium, and when in the early log phase, combined with top agar and used in the spot assay with the tenfold dilution of phage stock from the first part of the experiment. After 24 h of incubation (37 °C), the plates were examined for lysis zones or lysis-like zones. According to the presence and abundance of lysis zones, phage lifestyles were predicted. For each phage, at least 12 host strain variants, which were exposed to the phage, were tested.

### Genome and metavirome sequencing of phages and bacteria on the Illumina platform

Genomic phage DNA was extracted from stock solutions of phage lysates and fecal water samples. Samples (400 µL) were treated with 0.02 mg/mL DNAse I (Sigma Aldrich) and 0.05 mg/mL RNAse A (Qiagen) for 30 min at 37 °C. Enzymes were deactivated with DNAse stop solution (1 µL; Sigma Aldrich) and 10 min incubation at 70 °C. The DNA was then isolated using the NucleoSpin Virus kit (Macherey–Nagel) according to manufacturer’s instructions with prolonged incubations with proteinase K (30 min at 55 °C) and lysis buffer (15 min at 65 °C) to achieve better viral DNA quality. Additionally, the RTP^®^ DNA/RNA Virus Mini Kit (INVITEK Molecular) was used to isolate genomic DNA of a few samples^19^.

Bacterial DNA was extracted using the QIAamp DNA Mini Kit (Qiagen).

For phage and bacterial genomes, paired-end libraries were generated using the Nextera XT Library preparation kit (IIlumina) and sequenced on MiSeq (Ilumina) with 600-cycle MiSeq ReagentKit v3.

The quality of the raw sequencing reads was examined by FastQC tool Version 0.11.9 (Babraham Bioinformatics)^36^. Quality trimming was done by Trimmomatic Version 0.39 (USADELLAB.org)^37^ and overlapping paired-end reads were merged using FLASH software, version 1.2.11^38^. Assembly was performed by SPAdes Assembler (meta spades for metaviromes), version 3.14.0^39^, and the assemblies were examined using Quast version 4.0^40^. Core genome alignments of phages and bacterial genomes were generated with Roary^41^, phylogenetic trees were then generated with SeaView Version 5.0.2^42^ integrated phyML using the maximum likelihood approach (bootstrap value 1000) and the general time reversible (GTR) model nucleotide substitution model. The resulting dendrogram was then visualized with MEGA 11 software^43^.

Core genome analysis of *B. uniformis, Bacteroides ovatus, Phocaeiola vulgatus*, and *B. thetaiotaomicron* isolates also included approximately 10 phylogenetically diverse strains of each of the above species from the NCBI database (Supplementary Fig. 1). Additional core genome analyses of lineages with highly related bacterial strains were carried out for each developmental line, followed by determination of pairwise distances of bacterial strains expressed as SNPs^43^.

### Annotation and comparative analysis of phages

Protein-coding genes and tRNA were predicted and annotated with Prokka 1.14.5^44^. Protein sequences of open reading frames (ORFs) were then blasted (BLASTp, NCBI, 2019–2022)^23^ against the non-redundant protein sequences (nr) database. Conserved protein domains of ORFs were predicted with Conserved Domain Search (CDD, NCBI)^45^. Additionally, remote homologs were also detected using PHYRE2 (Protein Homology/analogY Recognition Engine) V 2.0^46^. Remote homologs of phage head-neck-tail module proteins were also analyzed on the VIRFAM server^47^. Predicted DGR regions were analyzed with myDGR^48^. Phage genome modules were determined manually based on predicted protein functions.

vConTACT2^21^ was used for taxonomic classification using the ViralRefSeq-prokaryotes-v94 database and protein coding sequences of known isolated gut phages from the literature.

In each phage cluster, a reference phage was chosen, and genetic differences inside the cluster were then analyzed by mapping the reads to the reference phage genome with BBtools^49^ and Samtools^50^. Single nucleotide polymorphisms (SNPs) were visualized and examined with Artemis software^51^. Genomic inversions were analyzed and detected with Genome Pair Rapid Dotter-Gepard^52^.

### Identification of prophage regions in isolated bacterial strains

Assemblies of sequenced bacterial strains from D1 and D2 (Supplementary Table 1) were used as a local database to screen for the presence of phages isolated in this study with blastn tool^23^. When putative prophage regions were detected, reads of original isolated phage genomes were mapped to the bacterial genome assembly using BBtools^49^. Sorted binary alignment map (BAM) files were used for calling SNP sites using Samtools^50^. Mapped reads and SNP sites were also analyzed using Artemis^51^, where intact complete prophage regions were extracted, annotated with Prokka^44^ and compared to other phages in the cluster.

### Identification of phages in metaviromes of fecal water samples

Assembled contigs in three metaviromes (D1-FW2, D2-FW1, and D2-FW2) were used as a local blastn database to screen for the presence of phages isolated in this study as well as gut phages described in the literature that infect dominant gut bacteria. Additionally, contigs larger than 20 kbp were blasted (blastn, NCBI) to obtain hits with the highest nucleotide similarity. Contigs that matched phages from isolated clusters were further annotated with Prokka^30^ and manually compared to isolated phages using Artemis genome editor.

## Supplementary Information

Supplementary Information 1.

Supplementary Information 2.

Supplementary Information 3.

Supplementary Information 4.

Supplementary Information 5.

Supplementary Information 6.

Supplementary Information 7.

## Data Availability

The datasets generated in this study can be found at the NCBI (https://www.ncbi.nlm.nih.gov/) under the Bioproject accession numbers PRJNA843113, PRJNA636979^19^ (bacterial genomes), and PRJNA638235 (phage genomes). *Bacuni* phage genomes are available under accession numbers: MT635598.1 (F1), MT806185.1 (F4), MT806186.1 (partial genome; F2), and MT806187.1 (partial genome; F2)^19^. The remaining phage genomes isolated in this study are available in Supplementary File 7 and will be available in the NCBI database after submission.
