# Supplementary material for: Broad host range may be a key to long-term persistence of bacteriophages infecting intestinal Bacteroidaceae species
Source: Sci Rep. 2022 Dec 6;12:21098. doi: 10.1038/s41598-022-25636-x (PMC9727126; doi:10.1038/s41598-022-25636-x)
Supplement: Supplementary file 1 — Supplementary Information 1. [file 41598_2022_25636_MOESM1_ESM.pdf]

List of supplementary files

Supplementary file 1 (this file): Supplementary Figures 1-4.

Supplementary file 2: Table S1. List of isolated bacterial strains.

Supplementary file 3: Table S2. Genome annotation of phage cluster representatives.

Supplementary file 4: Table S3. Phage representation in the obtained metaviromes.

Supplementary file 5: Table S4. Identification of contigs >20 kbp in the obtained metaviral samples.

Supplementary file 6: vConTACT2 analysis .cys file that can be opened using Cytoscape.

Supplementary file 7: Nucleotide sequences of isolated phage genomes.

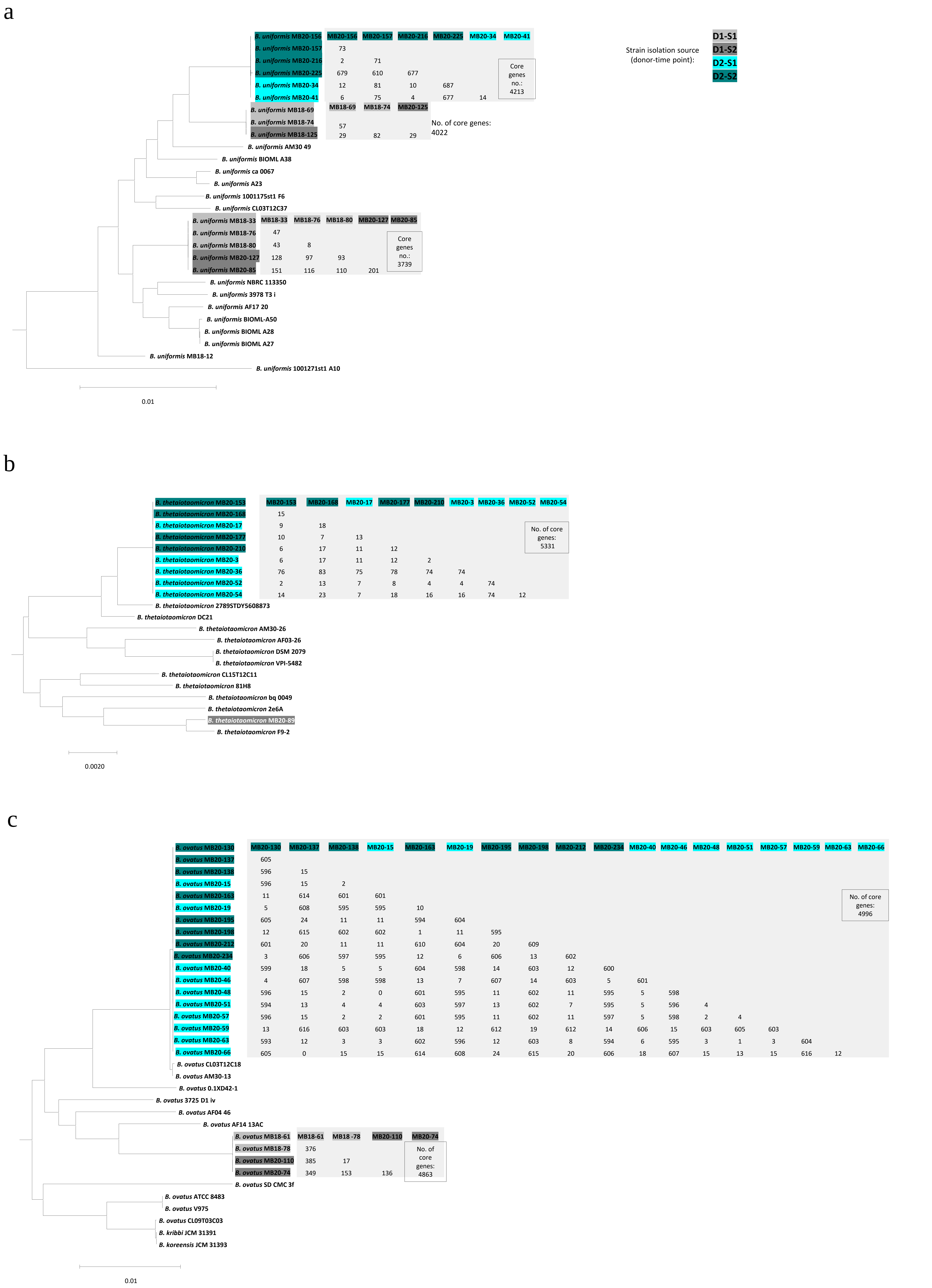

Supplementary Figure 1. Phylogenetic trees based on core-genome alignment of *B. uniformis* (a), *B. thetaiotaomicron* (b) and *B. ovatus* (c) strains. The genomic differences between strains clustered in one developmental line are given as number of SNP sites in the adjacent tables.

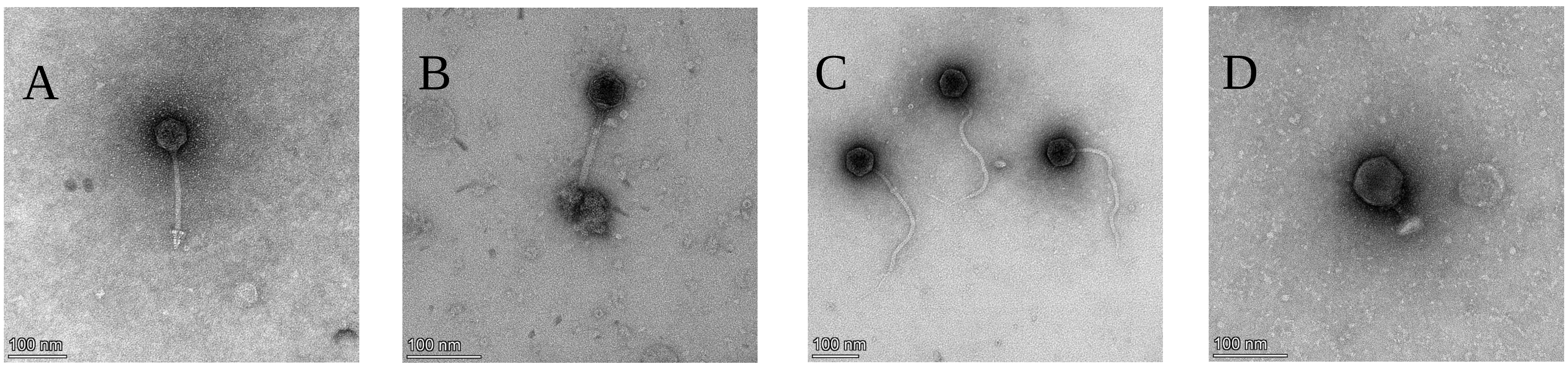

Transmission electron microscopy (TEM) revealed siphoviruses in the isolated and purified cultures of phages C1 (A), C2 (B) and C3 (C). Podovirus morphology was observed in TEM micrographs of D2-FW2 (D).

Transmission electron microscopy of cluster C1 representative, C1-85S2P, showed siphovirus morphology with a long, non-contractile tail and capsid diameter around 52 nm (A). The tail length (160 nm) is longer than in typical siphoviruses and ends in an arrow shaped tip. TEM of reference C2 phage, C2-80S2P, also exhibited siphovirus morphology with a 119 nm long tail and a capsid diameter around 51 nm (B). C3 reference phage C3-41T2LP again had siphovirus morphology with 57.5 nm capsid diameter and unusually long, bent tail, which together with a tail fibril measures 325 nm (C). Cluster C7 is Bacuni phage and the siphovirus morphology was described before[19]. TEM was additionally performed on fecal water sample D2-FW2, that was the isolation source of C4 phages (Crassphages). Phages of podovirus morphology (67 nm capsid diameter and 22 nm fibril length) were highly abundant (D).

E: Genetic and isolation differences within clusters

**Phage cluster**	**Nucleotide identity within cluster**	**No. of SNP sites**	**SNP locations in putative proteins**	**Isolation method; phage purification and plaque formation***	**Lysogeny associated genes****	**Detected as a prophage in isolated *Bacteroides* genomes**
C1	99%	3-9	Tail tape-measure protein, major capsid protein, portal protein; major tropism determinant	DAL; pure and mixed phage lysates; polymorphic plaques	No	no
C2	97-99%	4-255	Condensed SNPs sites in tail, capsid, coat, fimbrial, membrane domain proteins, putative packaging genes	DAL; pure and mixed phage lysates; polymorphic plaques	No	no
C3	99%	2-10, genome inversions	Major outer capsid, tail, membrane adhesion protein, envelope glycoprotein	DAL + enrichment; pure and mixed phage lysates, pure and mixed filtrates; slightly turbid 5 mm plaques	Yes	no
C4	99%	20	Portal protein, lysis regulatory protein, stabilization protein, peptidoglycan hydrolases	Enrichment culturing; mixed phage filtrates	No	no
C5	99%	n.a.	n.a.	Enrichment culturing; mixed phage filtrates	Yes	Yes; 7 in *P. vulgatus*, 1 in *B. uniformis*
C6	99%	n.a	n.a	DAL; mixed phage lysates;	Yes	Yes, strains from NCBI nr-database
C7	99%	9-27	Condensed in diversity generating element genes (DGR) especially in target protein DUF1566 (fimbrial tip protein)	DAL + enrichment; pure and mixed phage lysates; 3-0,5 mm plaques	Yes	Yes, 5 in *P. vulgatus*

*: DAL double agar layer methods; ** Putative integrase, repressor and excision genes.

F

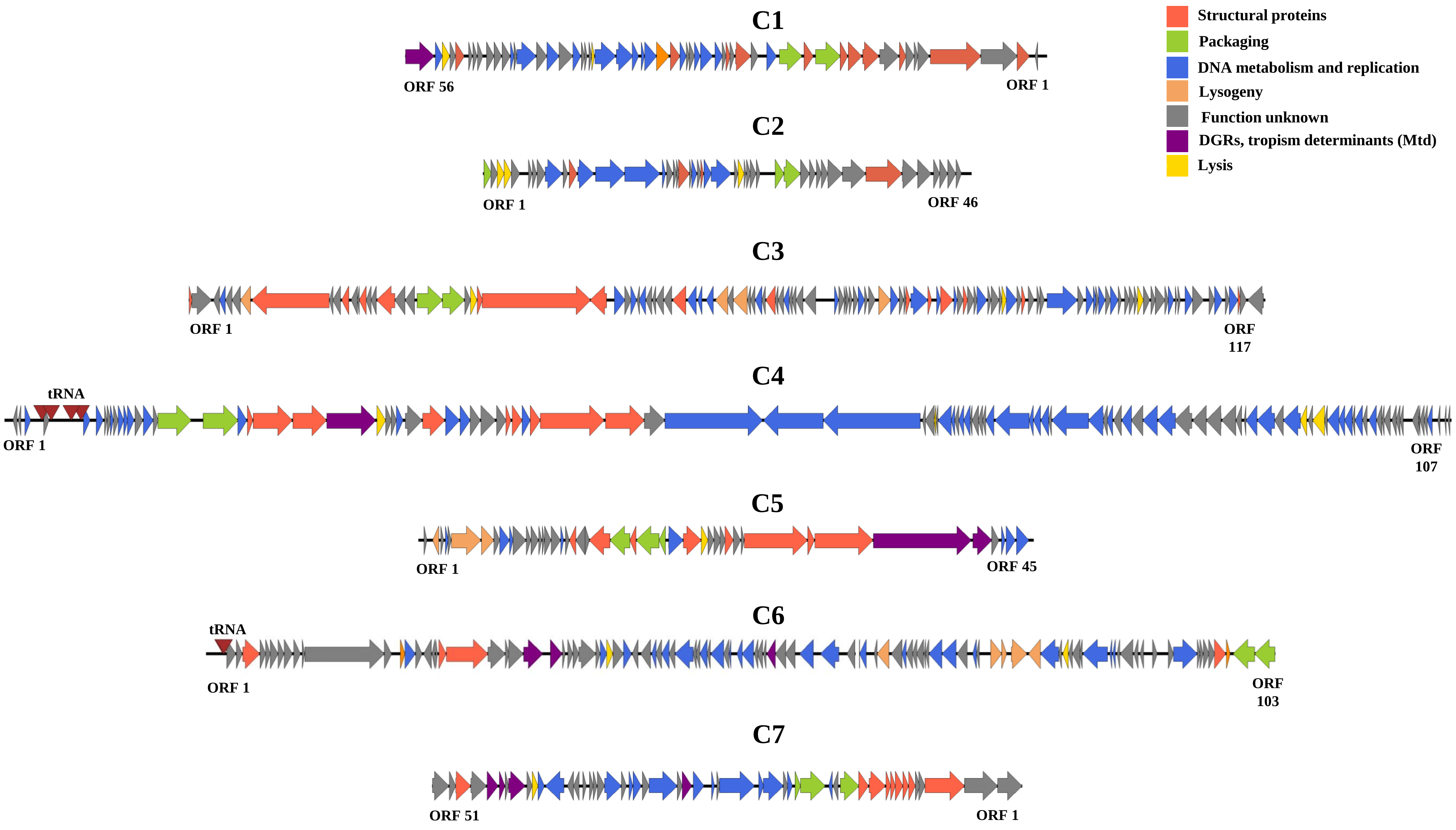

Supplementary Figure 2. Detailed characterization of isolated phages. A-D:Transmission electron microscopy. E: Genetic and isolation differences within clusters. F: Visualization of genome organization modules. ORF: open reading frame. There is little or zero protein homology between isolated bacteriophage clusters.

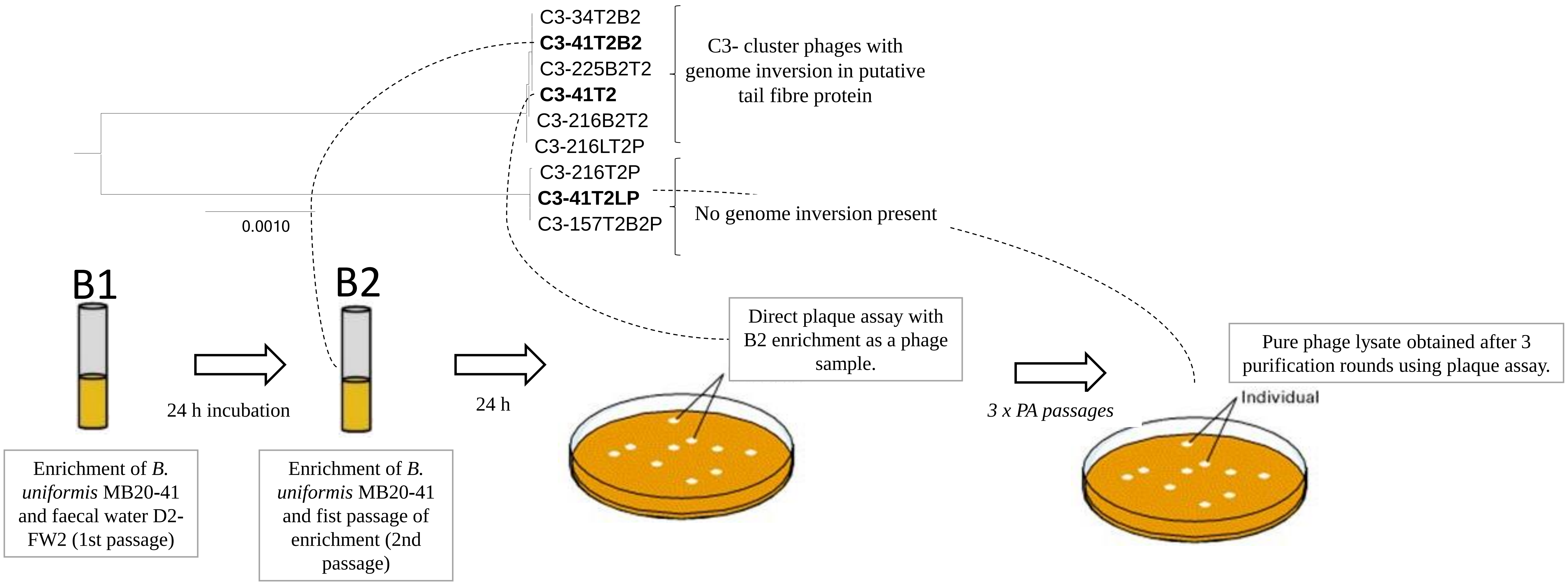

Supplementary Figure 3. Phylogenetic tree based on core genome alignment and isolation process of C3 phages. The phages with letter P in their names have all been purified by three rounds of plaque assay.

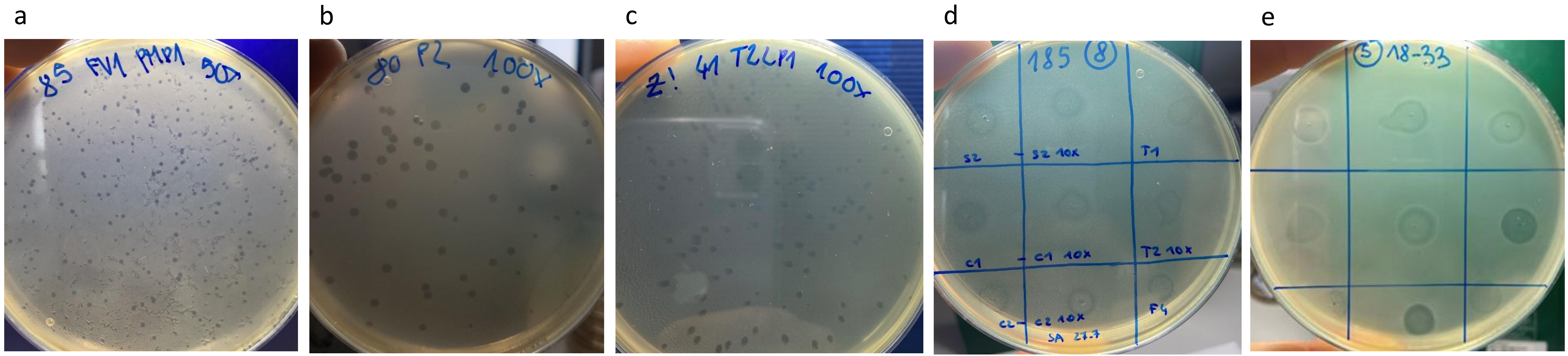

Supplementary figure 4. a-c: plaques of phages on bacterial lawns. Bacteriophage C1_85FV1PM on MB20-85 (a), C2_1880S2P on MB18-80 (b) and C3_41T2LP on MB20-41 (c). d: lysis-like zones on spot assay using strain MB20-185. Phages C1_85FV1PM and C2_1880S2P in middle and bottom rows as indicated. e: lysis-like zones and clearings on MB18-33. The clearings are caused by C7_F1 (bottom) and C1_85FV1PM (right).
