# Supplementary material for: Broad host range may be a key to long-term persistence of bacteriophages infecting intestinal Bacteroidaceae species
Source: Sci Rep. 2022 Dec 6;12:21098. doi: 10.1038/s41598-022-25636-x (PMC9727126; doi:10.1038/s41598-022-25636-x)
Supplement: Supplementary file 4 — Supplementary Information 4. [file 41598_2022_25636_MOESM4_ESM.docx]

Supplementary table 3: Phage representation in obtained metaviromes

| **Metavirome sample** | **D1-FW2** | **D2-FW1** | **D2-FW2** |
| --- | --- | --- | --- |
| No. of contigs above 20 kbps | 32 | 3 | 26 |
| Contig with highest coverage (nucleotide coverage/identity %) | Siphoviridae sp. isolate ct6At26 (BK018141.1) (27/94) | CrAssphage LMMB (MT006214.1) (95/94) (Morozova *et al.*, 2020) | Uncultured crAssphage (NC_024711.1) (95/97) (Dutilh *et al*., 2014) |
| Presence of phages isolated in this study (cluster name) | C1, C2, C6 and C7 phages | Partial C4 phage (74kb),  C5 phage | C3 cluster phage  Partial C4 phage (84 kbp),  C5 phage |
| Presence of described gut phages from literature | *F. prausnitzii* prophage Mushu (Cornuault *et al*., 2018);  Parabacteroides phage PDS1 (Guerin, 2020)  LoVEphage (Van Espen *et al*., 2021);  Bacuni (Hedžet *et al*., 2021) | CrAssphage LMMB (Morozova *et al.*, 2020); Bacteroides phage p00 Hankyphage (Benler *et al*., 2018) | *F. prausnitzii* and *Blautia hansenii* prophages Lagaffe and Mushu (Cornuault *et al*., 2018); Bacteroides phage p00 Hankyphage (Benler *et al*., 2018),  Uncultured crAssphage (Dutilh *et al*., 2014) |

Note: Sequencing of D1-FW1 did not result in successful assembly.

References:

Cornuault JK, Petit MA, Mariadassou M, et al. Phages infecting Faecalibacterium prausnitzii belong to novel viral genera that help to decipher intestinal viromes. *Microbiome*. 2018;6(1):65.. doi:10.1186/s40168-018-0452-1

Guerin, E. 2020. Mining for novel human gut bacteriophages against Bacteroidales. PhD Thesis, University College Cork.

Van Espen L, Bak EG, Beller L, et al. A Previously Undescribed Highly Prevalent Phage Identified in a Danish Enteric Virome Catalog. *mSystems*. 2021;6(5):e0038221. doi:10.1128/mSystems.00382-21

Hedžet S, Rupnik M, Accetto T. Novel *Siphoviridae* Bacteriophages Infecting *Bacteroides uniformis* Contain Diversity Generating Retroelement. *Microorganisms*. 2021;9(5):892. Published 2021 Apr 21. doi:10.3390/microorganisms9050892

Morozova V, Fofanov M, Tikunova N, Babkin I, Morozov V V, Tikunov A. First crAss-Like Phage Genome Encoding the Diversity-Generating Retroelement (DGR). Viruses. 2020;12(5). doi:10.3390/v12050573

Benler S, Cobián-Güemes AG, McNair K, et al. A diversity-generating retroelement encoded by a globally ubiquitous Bacteroides phage Microbiome. 2018;6(1):1-10. doi:10.1186/s40168-018-0573-6

Dutilh BE, Cassman N, McNair K, et al. A highly abundant bacteriophage discovered in the unknown sequences of human faecal metagenomes. Nat Commun. 2014;5:1-11. doi:10.1038/ncomms5498
